# Preoperative Inflammatory Markers (NLR, MLR, PLR) in Evaluating Acute Cholecystitis Severity and Operative Difficulty

**DOI:** 10.3390/life16040565

**Published:** 2026-03-30

**Authors:** Catalin Vladut Ionut Feier, Melania Veronica Ardelean, Calin Muntean, Alaviana Monique Faur, Vasile Gaborean, Marius Sorin Murariu

**Affiliations:** 1 Abdominal Surgery and Phlebology Research Center, “Victor Babeş” University of Medicine and Pharmacy Timişoara, 300041 Timişoara, Romania; catalin.feier@umft.ro (C.V.I.F.); murariu.marius@umft.ro (M.S.M.); 2 First Surgery Clinic, “Pius Brinzeu” Clinical Emergency Hospital, 300723 Timişoara, Romania; 3 Department V, Internal Medicine I, “Victor Babeş” University of Medicine and Pharmacy Timişoara, Eftimie Murgu Square 2, 300041 Timişoara, Romania; 4 Center of Advanced Research in Cardiology and Hemostasology, “Victor Babeş” University of Medicine and Pharmacy Timişoara, Eftimie Murgu Square 2, 300041 Timişoara, Romania; 5 Medical Informatics and Biostatistics, Department III-Functional Sciences, “Victor Babeş” University of Medicine and Pharmacy Timişoara, Eftimie Murgu Square No. 2, 300041 Timişoara, Romania; 6 Department of Doctoral Studies, “Victor Babeş” University of Medicine and Pharmacy Timişoara, 300041 Timişoara, Romania; alaviana.faur@umft.ro; 7 Thoracic Surgery Research Center, “Victor Babeş” University of Medicine and Pharmacy Timişoara, Eftimie Murgu Square No. 2, 300041 Timişoara, Romania; vasile.gaborean@umft.ro; 8 Department of Surgical Semiology, Faculty of Medicine, “Victor Babeş” University of Medicine and Pharmacy Timişoara, Eftimie Murgu Square No. 2, 300041 Timişoara, Romania

**Keywords:** acute cholecystitis, neutrophil-to-lymphocyte ratio, NLR, surgical conversion, gangrenous cholecystitis, inflammatory biomarkers, operative risk

## Abstract

Background: Acute cholecystitis, a leading cause of urgent surgical intervention, poses challenges in predicting severity and operative complexity. This study characterized the immuno-inflammatory profile distinguishing acute from chronic cholecystitis and assessed whether blood-derived ratios—neutrophil-to-lymphocyte (NLR), monocyte-to-lymphocyte (MLR), and platelet-to-lymphocyte (PLR)—correlate with histologic severity and surgical difficulty. Methods: The study retrospectively analyzed 759 patients undergoing cholecystectomy from 2016 to 2024. Inflammatory indices from preoperative bloodwork were compared across histopathologic subtypes (catarrhal, phlegmonous, gangrenous), clinical features, and surgical outcomes, including conversion to open procedure. Logistic regression and ROC analyses identified predictors of acute inflammation and conversion. Results: Acute cholecystitis patients showed elevated NLR (7.0 vs. 3.1), MLR (0.44 vs. 0.26), and PLR (194 vs. 142; all *p* < 0.001). NLR was the only independent predictor of acute disease (OR = 1.29, 95% CI 1.203–1.390, *p* < 0.001), with superior discrimination (AUC = 0.806, cut-off = 3.56; sensitivity 73.1%, specificity 80.4%). NLR and PLR rose progressively from catarrhal to phlegmonous and gangrenous subtypes (*p* < 0.05), mirroring conversion rates (0% catarrhal, 3.2% phlegmonous, 10.5% gangrenous; *p* = 0.001). Conclusions: Routine hematologic ratios capture systemic immune activation in acute cholecystitis, reflecting histologic severity and operative risk. NLR, integrating innate and adaptive immune dynamics, offers a practical biomarker for preoperative risk stratification in acute care surgery.

## 1. Introduction

Acute cholecystitis remains one of the most frequent causes of emergency abdominal surgery worldwide. Epidemiological data indicate that more than 700,000 cholecystectomies are performed annually in the United States, making it the most common biliary surgical procedure [1]. In the United Kingdom, approximately 60,000 cholecystectomies are carried out each year, while European data estimate over 200,000 procedures annually in Germany alone [2,3]. The Tokyo Guidelines 2018 (TG18) provide the global standard for severity grading—Grade I (mild: local inflammation), Grade II (moderate: WBC > 18 × 10^9^/L, palpable mass), Grade III (severe: organ dysfunction)—strongly predicting 30-day mortality (0.5% vs. 15%), length-of-stay, and conversion rates (10–30% Grade II/III) [4]. However, TG18’s reliance on heterogeneous clinical signs and delayed organ failure limits preoperative risk stratification for surgical planning in emergency settings. Despite advances in preoperative evaluation, imaging modalities, and biochemical assessment, the accurate differentiation between moderate and severe forms of acute cholecystitis continues to pose a clinical challenge. This limitation is of particular importance in predicting the technical difficulty of cholecystectomy and optimizing surgical strategy to minimize intraoperative and postoperative risks [5].

The disease encompasses a continuous spectrum of inflammatory changes, ranging from simple edematous or catarrhal inflammation to phlegmonous and, ultimately, gangrenous destruction of the gallbladder wall. This pathological progression reflects the combined effects of luminal obstruction, ischemia, and bacterial translocation, which initiate a local cascade of cytokine-mediated inflammation and microvascular compromise [6]. Beyond these local mechanisms, the systemic immune response of the host plays a critical role, as the activation of neutrophils, macrophages, and platelets orchestrates both tissue injury and subsequent reparative processes [7].

In recent years, growing emphasis has been placed on elucidating the inflammatory and immunologic mechanisms underlying acute cholecystitis. Although the local pathophysiology is well established—namely cystic duct obstruction leading to bile stasis, increased intraluminal pressure, and subsequent bacterial proliferation—the systemic component of the disease has gained increasing clinical relevance [5,8]. The systemic impact is primarily mediated by activation of the innate immune system and the release of proinflammatory cytokines such as interleukin (IL)-6, IL-8, and tumor necrosis factor alpha (TNF-α), which together amplify the inflammatory cascade [7,9,10]. These mediators stimulate neutrophil mobilization, enhance monocyte adhesion, and promote lymphocyte redistribution, resulting in significant alterations in circulating leukocyte subsets and their functional capacity [11,12]. Consequently, the hematologic profile of patients with acute cholecystitis can be viewed as a dynamic reflection of the immune processes taking place within the gallbladder wall and, more broadly, of the systemic host response to localized inflammation [9,11].

Routine laboratory parameters such as white blood cell count and C-reactive protein remain the most commonly used tools for assessing the severity of acute cholecystitis; however, they provide only limited insight into the dynamic progression and immunological complexity of the disease [13]. In contrast, inflammatory markers such as the neutrophil-to-lymphocyte ratio (NLR), monocyte-to-lymphocyte ratio (MLR), and platelet-to-lymphocyte ratio (PLR) have emerged as reliable indicators of systemic inflammation [14]. Their value lies in integrating multiple immune cell lines, thereby offering a broader reflection of the balance between innate and adaptive immunity. Among these, elevated NLR has been most consistently associated with acute inflammatory and ischemic conditions—including appendicitis, pancreatitis, and cardiovascular events—highlighting its role as a universal biomarker of immune activation. Notably, these indices have also demonstrated diagnostic and prognostic utility in a wide range of other pathologies, including malignancies, cardiovascular diseases, and systemic inflammatory disorders, supporting their broader clinical applicability [15,16,17].

In biliary disease, several clinical investigations have demonstrated that elevated NLR values correlate with more severe or complicated forms of acute cholecystitis, a higher probability of conversion to open cholecystectomy, and less favorable postoperative recovery [16,18,19]. Reported cut-off thresholds and predictive accuracies, however, have varied considerably between studies, likely reflecting heterogeneity in study design, population demographics, and diagnostic criteria. Recent systematic reviews and meta-analyses have confirmed the diagnostic and prognostic value of NLR in acute cholecystitis, while also highlighting significant heterogeneity in reported thresholds and performance across studies [18,19,20]. However, MLR remains critically underexplored despite monocytes’ pivotal role in biliary immunothrombosis: IL-6/TNF-α-activated monocytes amplify cytokine storms, sustain neutrophil recruitment, and drive mural fibrosis, distinguishing TG18 progression. PLR similarly captures platelet-monocyte aggregates central to microvascular occlusion, yet no comparative analyses exist across all three ratios with matched controls, symptom correlation (fever/nausea), or comprehensive operative outcomes. Consequently, the broader clinical and immunologic relevance of these hematologic indices in gallbladder inflammation has not been fully elucidated.

From an immunologic standpoint, the coordinated activity of neutrophils, lymphocytes, monocytes, and platelets constitutes the cellular backbone of the systemic inflammatory response. Neutrophils act as rapid-response effectors, releasing proteolytic enzymes and reactive oxygen species that compromise epithelial integrity. Concurrent lymphocyte depletion attenuates immune regulation and perpetuates tissue injury, while activated platelets and monocytes contribute to immunothrombosis and endothelial dysfunction [12,21]. The composite ratios derived from these cell lines—NLR, MLR, and PLR—thus condense complex immune interactions into quantifiable parameters that reflect both the magnitude and balance of systemic inflammation.

By combining routine hematologic markers with clinical findings and tissue pathology, our study explores the connection between immune imbalance and the challenges of surgery in acute cholecystitis. Pinpointing this link holds promise on two fronts: it may enable earlier spotting of at-risk cases, even prior to imaging, and guide surgeons on expected operative hurdles, the likelihood of switching to open surgery, and recovery risks.

We reviewed records from 759 cholecystectomy patients treated over nine years to clarify how systemic inflammation shapes the full range of cholecystitis, from mild to severe. In particular, we examined correlations between NLR, MLR, and PLR with symptoms at presentation, histologic grade, and surgery details. These ratios reliably separated acute from chronic cases and escalated from phlegmonous to gangrenous stages, signaling tougher operations and more open conversions through broader immune overdrive. In essence, we tested whether these accessible blood tests could serve as everyday aids for sharper diagnosis, patient risk grouping, and better operating room prep. At its core, the work posits that rising NLR, MLR, and PLR levels track both the fervor of the immune response and the real-world demands of gallbladder surgery.

## 2. Materials and Methods

This retrospective observational study included patients who underwent cholecystectomy at the First Surgery Clinic of the Pius Brînzeu County Emergency Clinical Hospital in Timișoara, Romania—a tertiary referral center. Data were collected over a nine-year period, from 1 January 2016 to 31 December 2024.

Eligible cases were identified according to predefined inclusion criteria. Records were reviewed for adult patients (>18 years) who underwent surgery for either acute or chronic calculous cholecystitis during the study period. Because several conditions are known to significantly influence systemic inflammatory responses, patients with diabetes, autoimmune diseases, and malignancy were excluded. In particular, as cancer profoundly alters inflammatory markers [22,23,24], patients with active malignancy, previous oncologic history, or those who had received chemotherapy, radiotherapy, or immunotherapy were not included in the study.

Histopathological reports were used to confirm postoperative diagnoses. Patients with acute calculous cholecystitis were subclassified into catarrhal, phlegmonous, and gangrenous forms. This classification was based on standard microscopic criteria, including the extent of inflammatory infiltrate, degree of mural edema, and the presence of ischemia or transmural necrosis. Catarrhal cholecystitis was defined by mild inflammatory changes, phlegmonous by marked neutrophilic infiltration and wall thickening, and gangrenous by the presence of transmural necrosis. To minimize potential selection bias and ensure balanced demographic representation, each acute case was matched with two distinct patients who had undergone surgery for chronic calculous cholecystitis, based on age and sex. Each chronic control was assigned to a single acute case and was not reused across different histopathologic subgroups.

This matching strategy resulted in a 1:2 ratio (acute:chronic) for catarrhal, phlegmonous, and gangrenous cholecystitis, while maintaining non-overlapping control groups. By design, this approach standardized comparisons and reduced confounding effects related to demographic imbalance.

After applying all inclusion and exclusion criteria, a total of 759 patient records were analyzed. For each case, demographic variables (sex, age, and rural vs. urban origin) were documented. The preoperative hematologic parameters included absolute counts of lymphocytes (Lym), monocytes (Mon), neutrophils (Neu), and platelets (Pla). Blood samples were routinely collected at admission, as part of the initial clinical and laboratory assessment, prior to the initiation of any therapeutic interventions. Derived inflammatory indices were calculated as follows:

• Neutrophil-to-lymphocyte ratio (NLR) = Neu/Lym;
• Monocyte-to-lymphocyte ratio (MLR) = Mon/Lym;
• Platelet-to-lymphocyte ratio (PLR) = Pla/Lym.

Clinical variables assessed at admission included the presence of nausea, vomiting, fever, and abdominal pain. Operative details were also recorded, specifying whether the procedure was completed laparoscopically or required conversion to an open approach.

The study was conducted in accordance with the principles of the Declaration of Helsinki and approved by the Ethics Committee of the “Pius Brînzeu” Emergency County Clinical Hospital, Timișoara, Romania (Approval No. 551/22.07.2025). Given the retrospective design and use of anonymized data, the requirement for informed consent was waived in line with institutional and national regulations.

### Statistical Analysis

The normality of distribution was evaluated using the Shapiro–Wilk test, while homogeneity of variances was verified by Levene’s test. Continuous variables with normal distribution were compared using the independent-samples *t*-test, whereas non-normally distributed data were analyzed using the Mann–Whitney U test. Descriptive statistics were reported as means ± standard deviations (SD) for continuous data and as frequencies (*n*, %) for categorical variables. Between-group comparisons were performed using the independent-samples *t*-test for normally distributed data and the Mann–Whitney U test for non-normally distributed data. For comparisons involving more than two groups, one-way ANOVA or the Kruskal–Wallis test was applied, as appropriate, followed by post hoc analyses with Bonferroni correction. Categorical variables were analyzed using the Chi-square or Fisher’s exact test, as appropriate.

Binary logistic regression was used to identify independent associations between systemic inflammatory indices (NLR, PLR, and MLR) and the presence of gangrenous cholecystitis. Odds ratios (OR) with 95% confidence intervals (CI) were calculated. Receiver operating characteristic (ROC) curve analysis was conducted to evaluate the discriminative ability of each index, with area under the curve (AUC) and optimal cut-off points determined via Youden’s Index. Sensitivity, specificity, and likelihood ratios were reported for each threshold. A *p*-value < 0.05 was considered statistically significant. All analyses were performed using Jamovi software (version 2.6.26.0), and figures were generated using Microsoft Excel and Jamovi.

## 3. Results

A total of 759 patients who underwent cholecystectomy at the First Surgery Clinic of the Pius Brînzeu County Emergency Clinical Hospital in Timișoara, Romania, between 1 January 2016 and 31 December 2024, were included in this study.

### 3.1. Key Information

Among the 759 patients analyzed, 687 (91.5%) underwent laparoscopic cholecystectomy, 37 (4.9%) were operated using the open approach, and conversion from laparoscopy to open surgery was required in 27 cases (3.6%).

The open approach was significantly more frequent in patients with acute cholecystitis compared to those with chronic disease (7.7% vs. 3.9%, *p* = 0.028). Similarly, the conversion rate from laparoscopic to open surgery was higher in the acute group (6.0% vs. 2.4%, *p* = 0.004).

Demographic characteristics of the study population are summarized in Table 1.

Abdominal pain represented the most frequent presenting symptom, reported in 95.8% of all patients, and occurred significantly more often among those with acute cholecystitis compared to chronic cases (98.4% vs. 94.5%, *p* = 0.011).

Nausea and vomiting were likewise more prevalent in the acute group (83.4% vs. 72.7%, *p* = 0.001; and 68.4% vs. 51.0%, *p* < 0.001, respectively). Fever was documented in 7.5% of patients overall, with a markedly higher incidence in the acute cohort (13.8% vs. 4.3%, *p* < 0.001).

The comparative variation in inflammatory markers between the two groups is detailed in Table 2.

Regarding the presence of nausea at admission, no significant difference in patient age was identified between those presenting with and without this symptom (58.79 ± 14.72 years vs. 59.52 ± 14.89 years, *p* = 0.562). However, nausea was significantly more frequent among female patients (79.2% vs. 71.5%, *p* = 0.018). Among inflammatory parameters, patients reporting nausea showed significantly higher neutrophil counts (6.94 ± 4.15 vs. 5.84 ± 3.54, *p* = 0.003) and elevated NLR values (4.63 ± 3.55 vs. 3.55 ± 2.57, *p* = 0.006), while MLR exhibited a nonsignificant upward trend (0.34 ± 0.26 vs. 0.28 ± 0.17, *p* = 0.070). Other parameters showed no statistically significant variation.

Similarly, for vomiting at admission, no age-related differences were observed (58.85 ± 14.44 years vs. 59.10 ± 15.18 years, *p* = 0.814), but the symptom was more prevalent in females (59.7% vs. 52.1%, *p* = 0.042). Significant increases were recorded in total neutrophil count (7.24 ± 4.33 vs. 5.96 ± 3.53, *p* < 0.001), NLR (4.86 ± 3.38 vs. 3.76 ± 2.27, *p* = 0.001), and PLR (165.56 ± 93.15 vs. 151.85 ± 93.50, *p* = 0.045).

Regarding fever, age did not differ significantly between febrile and afebrile patients (61.79 ± 14.09 years vs. 58.73 ± 14.79 years, *p* = 0.132), and no sex-based difference was identified (49.1% vs. 50.9%, *p* = 0.088). Inflammatory markers, however, showed significant alterations in all parameters except platelet count (273.34 ± 104.66 vs. 268.00 ± 84.74, *p* = 0.654). Patients with fever exhibited lower lymphocyte counts (1.63 ± 0.81 vs. 1.99 ± 0.77, *p* = 0.001) and higher values for monocytes (0.63 ± 0.41 vs. 0.53 ± 0.35, *p* = 0.045), neutrophils (9.38 ± 5.38 vs. 6.46 ± 3.84, *p* < 0.001), NLR (8.02 ± 5.99 vs. 4.08 ± 3.94, *p* < 0.001), MLR (0.51 ± 0.39 vs. 0.31 ± 0.21, *p* < 0.001), and PLR (217.53 ± 148.46 vs. 154.93 ± 86.0, *p* < 0.001).

### 3.2. Predictive and Diagnostic Value of NLR, PLR and MLR in Acute Cholecystitis

To assess the independent predictive value of the inflammatory indices included in the study, a binary logistic regression analysis was performed, using the type of cholecystitis (acute vs. chronic) as the dependent variable and the NLR, MLR, and PLR values as independent variables. The model aimed to identify which markers were significantly associated with the presence of the acute form. In the logistic regression model, where all the 3 ratios were analyzed, NLR was independently associated with acute cholecystitis (OR = 1.293, 95% CI, *p* < 0.001), indicating a 29% increase in odds for each unit increase. MLR and PLR did not reach statistical significance in predicting acute disease. Results are presented in Table 3.

**Figure 1:**
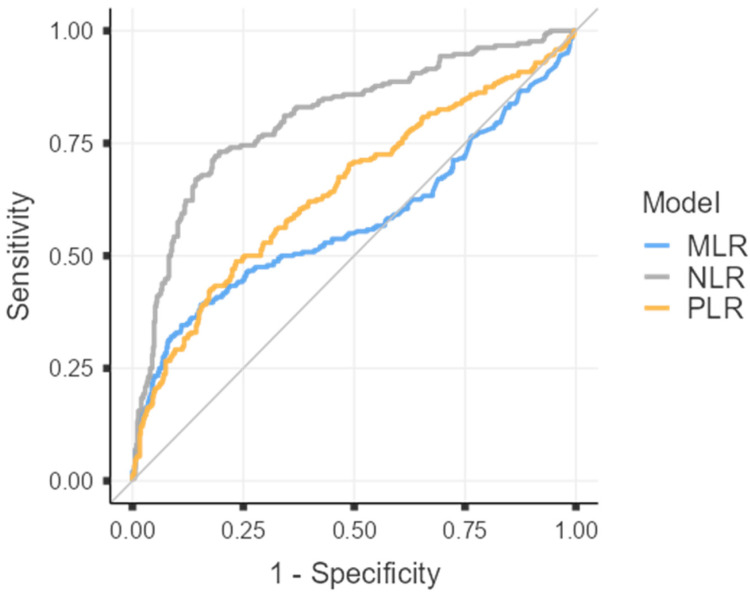
Combined receiver operating characteristic.

To evaluate the discriminative capacity of each inflammatory index in differentiating acute from chronic cholecystitis, receiver operating characteristic (ROC) curves were generated for the three analyzed parameters, as illustrated in Figure 1.

The variation in the area under the curve (AUC) values for the three analyzed inflammatory parameters is presented in Table 4, highlighting distinct predictive performances among them. NLR achieved the highest AUC, indicating strong discriminative ability between acute and chronic forms of cholecystitis, while MLR and PLR exhibited lower AUC values, suggesting limited diagnostic precision.

Among the three investigated markers, only NLR demonstrated a statistically significant potential for discriminating between acute and chronic forms of cholecystitis. The ROC curve analysis confirmed its predictive value, with an area under the curve (AUC) of 0.806, compared to 0.609 for MLR and 0.666 for PLR. Given the excellent discriminative ability of NLR (AUC > 0.8), an optimal cut-off value was determined to distinguish acute from chronic disease. A threshold of 3.56 was identified, providing a sensitivity of 73.11%, a specificity of 80.44%, and an overall diagnostic accuracy of 78.10%. The detailed statistical analysis and corresponding results are summarized in Table 5 and Table 6.

### 3.3. Acute Cholecystitis Types

Regarding the distribution of acute cholecystitis types, 13 patients (5.1%) presented with catarrhal cholecystitis, 95 (37.5%) with phlegmonous cholecystitis, and 145 (57.3%) with the gangrenous form. A significant increase (*p* = 0.001) in the conversion rate from laparoscopic to open surgery was observed with advancing severity. All patients with catarrhal cholecystitis were managed laparoscopically, while conversion was required in 3 cases (3.2%) of phlegmonous and in 15 cases (10.5%) of gangrenous cholecystitis.

Given the small number of catarrhal cases (*n* = 13) and to minimize bias, subsequent analyses focused on the inflammatory profile of phlegmonous and gangrenous cholecystitis.

Among the 95 patients with phlegmonous cholecystitis, results were compared with those of 190 patients with chronic disease. No significant differences were found in monocyte count (0.53 ± 0.39 vs. 0.54 ± 0.18 × 10^9^/L, *p* = 0.661), platelet count (272.29 ± 89.27 vs. 277.5 ± 76.0 × 10^9^/L, *p* = 0.608), or MLR (0.38 ± 0.29 vs. 0.29 ± 0.28, *p* = 0.058). In contrast, significant elevations were noted for NLR (5.77 ± 4.33 vs. 3.83 ± 2.31, *p* = 0.002) and PLR (172.52 ± 97.14 vs. 146.31 ± 63.96, *p* = 0.007).

The differences became more pronounced in the gangrenous group (*n* = 145) compared with chronic cholecystitis (*n* = 290), where all investigated parameters showed statistically significant deviations. The detailed results are presented in Table 7.

Lastly, we aimed to determine whether significant differences existed between phlegmonous and gangrenous acute cholecystitis by analyzing variations in inflammatory markers. No statistically significant differences were found in total monocyte count (0.53 ± 0.39 vs. 0.65 ± 0.53 × 10^9^/L, *p* = 0.055), platelet count (272.29 ± 89.27 vs. 274.55 ± 107.6 × 10^9^/L, *p* = 0.876), or MLR (0.38 ± 0.29 vs. 0.49 ± 0.40, *p* = 0.098). In contrast, significant differences were observed in total lymphocyte and neutrophil counts, as well as in NLR and PLR values, all of which were elevated in gangrenous compared to phlegmonous forms. The detailed comparisons are presented in Table 8.

## 4. Discussion

This study provides new evidence on the interplay between systemic inflammation and surgical outcomes in cholecystitis, showing that hematologic indices derived from standard blood tests—particularly the neutrophil-to-lymphocyte ratio (NLR), monocyte-to-lymphocyte ratio (MLR), and platelet-to-lymphocyte ratio (PLR)—mirror the immune activation state and predict both disease severity and operative difficulty. By analyzing 759 consecutive patients who underwent cholecystectomy between 2016 and 2024, we found a strong immunologic and clinical pattern that distinguishes acute from chronic inflammation and correlates with histologic progression from phlegmonous to gangrenous cholecystitis.

### 4.1. Systemic Immune Imbalance

The acute form of cholecystitis displayed a distinct hematologic signature consistent with systemic inflammatory activation. Patients in the acute group showed markedly elevated neutrophil counts (9.3 vs. 5.4 × 10^9^/L, *p* < 0.001), lower lymphocyte levels (1.7 vs. 2.1 × 10^9^/L, *p* < 0.001), and substantially higher inflammatory ratios—NLR 7.0 vs. 3.1, MLR 0.44 vs. 0.26, and PLR 194 vs. 142 (all *p* < 0.001). This constellation mirrors an innate-dominant immune response characteristic of acute bacterial and ischemic tissue injury. Under the stimulation of proinflammatory cytokines such as IL-6 and TNF-α, neutrophils are rapidly mobilized, releasing proteolytic enzymes and reactive oxygen species that exacerbate mucosal damage [9,14]. In parallel, stress-induced lymphopenia occurs through glucocorticoid-mediated apoptosis and redistribution of lymphocytes [21,25]. Collectively, these mechanisms culminate in an imbalance between innate and adaptive immunity.

The NLR therefore serves as a compact, quantitative reflection of this dual process—capturing both the escalation of innate immune activity and the suppression of adaptive responses. This interpretation aligns with current evidence suggesting that NLR functions as an integrated biomarker of inflammatory stress across multiple organ systems [14,25]. In our regression model, NLR emerged as the sole independent predictor of acute disease (OR 1.29, *p* < 0.001), indicating that each unit increase in NLR corresponds to a 29% higher likelihood of acute cholecystitis. These findings reinforce its role as a sensitive and biologically meaningful index of immune activation in biliary inflammation.

The ROC curve analysis demonstrated that among all evaluated indices, NLR had the highest diagnostic performance, with an area under the curve (AUC) of 0.806, compared to 0.609 for MLR and 0.666 for PLR. The optimal NLR cut-off of 3.56 distinguished acute from chronic cholecystitis with 73.1% sensitivity and 80.4% specificity, achieving an overall accuracy of 78.1%. This level of discriminative power surpasses that of total leukocyte or C-reactive protein values reported in previous studies and confirms that simple cell-derived ratios can act as robust surrogates for more complex inflammatory markers. The findings are consistent with the results of other studies that identified similar NLR thresholds around 3.5–4.3 for severe or complicated cholecystitis [20,26].

These results emphasize that NLR, by integrating neutrophil proliferation and lymphocyte depletion, captures the systemic inflammatory burden more effectively than conventional laboratory markers. Its diagnostic superiority likely stems from its ability to mirror both the innate immune surge and the concurrent suppression of adaptive responses—a hallmark of acute inflammatory stress. Moreover, the reproducibility of this cut-off across independent cohorts strengthens its external validity and potential for clinical translation.

Studies recently demonstrated comparable AUC values (0.79) and optimal NLR thresholds near 3.8 for differentiating complicated from uncomplicated cholecystitis, supporting its use as a rapid screening metric in emergency settings [26]. Similarly, Uludağ et al. confirmed that elevated NLR correlates not only with disease severity but also with a higher probability of conversion to open surgery [20]. Collectively, these convergent data suggest that NLR may serve as a low-cost, easily measurable indicator capable of refining diagnostic algorithms and improving preoperative risk stratification in acute biliary inflammation [14,19,20,25,26,27].

### 4.2. Correlation Between Inflammatory Status and Symptoms

Systemic inflammatory activation closely mirrored the patients’ clinical presentation. Those presenting with nausea and vomiting exhibited significantly higher neutrophil counts and NLR values, whereas the presence of fever—a cardinal manifestation of cytokine-mediated immune activation—was accompanied by substantial elevations across all inflammatory ratios (NLR 8.0, MLR 0.51, PLR 217; all *p* < 0.001). These associations underscore the interplay between systemic immune activation and symptom burden, suggesting that hematologic indices dynamically reflect the clinical expression of inflammation [14,28,29].

Cytokines such as interleukin (IL)-1β and IL-6, key mediators of fever and malaise, promote both neutrophil mobilization and platelet activation, thereby altering peripheral cell ratios in a quantifiable manner [7,8,26]. The resulting hematologic profile, integrating these immune-driven shifts, serves not only as a marker of systemic inflammatory load but also as a surrogate for symptom intensity and physiologic stress [11,30,31].

Within the acute subgroup, our findings demonstrated a clear stepwise escalation of systemic immune activation paralleling histopathological severity. In phlegmonous cholecystitis, mean NLR reached 5.77 and PLR 172, while in gangrenous cases these values increased to 8.1 and 208, respectively (*p* < 0.05 for both). Lymphocyte counts declined further, reflecting progressive exhaustion of the adaptive response.

This gradient delineates an amplification loop of inflammation, in which persistent neutrophil activation, cytokine overproduction, and platelet–leukocyte interaction sustain endothelial injury and microvascular occlusion [9,11,21]. These intertwined mechanisms culminate in ischemic necrosis of the gallbladder wall—the histopathologic hallmark of gangrenous cholecystitis.

The observed hematologic escalation mirrors the process of immunothrombosis, whereby activated immune and hemostatic pathways jointly mediate pathogen containment but also amplify tissue damage [11,21,29]. Consequently, the peripheral inflammatory profile serves as a measurable systemic surrogate of the local immunopathology driving gallbladder necrosis.

### 4.3. Inflammatory Status and Surgical Complexity

The intensity of systemic inflammation translated directly into operative complexity and outcomes. Conversion to open cholecystectomy was necessary in 3.6% of all cases, but the rate increased steeply with histologic severity—0% in catarrhal, 3.2% in phlegmonous, and 10.5% in gangrenous cholecystitis (*p* = 0.001). This progression reinforces the association between immune activation and surgical difficulty, suggesting that systemic inflammatory load can predict intraoperative challenges.

Elevated NLR and PLR values likely reflect tissue edema, friability, and dense adhesions that obscure the critical view of safety and increase the risk of biliary injury [10,19,28]. Similar findings have been reported, which showed that NLR values exceeding 3.5 were strongly associated with prolonged operative time and higher conversion rates [32,33]. Recognizing this relationship allows surgeons to anticipate complexity, plan early conversion when appropriate, and mitigate complications through preoperative risk optimization.

From a practical perspective, integrating inflammatory ratios into surgical decision-making offers a rapid and objective tool for stratifying operative risk. Routine assessment of NLR, MLR, and PLR before surgery could support prioritization of difficult cases, guide allocation of experienced teams, and improve operative safety [19,20,28,32,33].

### 4.4. Clinical Implications and Future Perspectives

The immunologic and surgical correlations identified in this study reinforce the growing body of evidence that elevated NLR and PLR values predict not only the presence of complicated cholecystitis but also technical difficulty and postoperative morbidity. Recent studies have shown that higher NLR values are independently associated with prolonged operative time, increased intraoperative blood loss, and conversion to open cholecystectomy in acute disease [26,32,34].

Mechanistically, this association can be traced to cytokine-mediated interactions between immune and hemostatic systems. Activated neutrophils and platelets form aggregates through adhesion molecules such as P-selectin and β_2_-integrins, triggering the release of proteolytic enzymes and neutrophil extracellular traps (NETs) that intensify local thrombosis and fibrosis [11,21,35,36]. This immune–hemostatic crosstalk, while essential for pathogen containment, leads to stromal stiffness and adhesions that complicate the operative field.

Our findings support this continuum between systemic inflammation and surgical complexity, highlighting how the biological processes quantified in the bloodstream mirror the mechanical challenges encountered intraoperatively. In this context, NLR and related indices represent not only markers of immune imbalance but also predictors of operative behavior and outcome [26,32,34,35,36,37].

#### Study Limitations

This retrospective single-center investigation, conducted at a high-volume Romanian tertiary referral center, imposes inherent constraints on external validity, as local demographic profiles, antimicrobial stewardship practices, and operative protocols may diverge from those in heterogeneous international cohorts. Although the 1:2 matching scheme across catarrhal: chronic, phlegmonous: chronic, and gangrenous: chronic subgroups effectively controlled for age and sex disparities while enabling precise delineation of inflammatory gradients, residual confounders—including undocumented pre-operative antibiotic administration, temporal discrepancies between symptom onset and occult comorbidities such as subclinical metabolic dysregulation—may subtly modulate hematologic indices and preclude definitive causal inferences. The retrospective design of this study represents a major limitation, as it inherently restricts control over confounding variables and the completeness of clinical data. Histopathological subcategorization, despite reliance on standardized microscopic criteria, remains susceptible to inter-observer variability in assessing necrosis depth, inflammatory infiltrate density, or fibrotic remodeling, potentially attenuating the observed progression from phlegmonous to gangrenous forms. Furthermore, involvement of multiple surgeons across the study interval introduces heterogeneity in laparoscopic proficiency, which could independently elevate conversion rates beyond inflammation-driven tissue friability. The lack of formal calibration and external validation represents a limitation of the present study. Nonetheless, the cohort’s scale (759 patients) and comprehensive acute–chronic–histologic stratification establish a robust foundation for subsequent multicenter prospective validation.

## 5. Conclusions

This study establishes that routine blood-derived inflammatory indices, particularly NLR, robustly distinguish acute from chronic cholecystitis and outperform MLR/PLR. NLR’s escalation from phlegmonous to gangrenous forms parallels immunologic progression—from innate surge to adaptive exhaustion—with histologic necrosis and surgical complexity. Mechanistically linked to neutrophil-driven immunothrombosis, these markers offer prognostic value for operative planning. Integrating NLR into preoperative assessment promises refined risk stratification and optimized outcomes in acute biliary inflammation.

## Figures and Tables

**Table 1:** Demographic characteristics.

Characteristic	All (*n* = 759)	Acute (*n* = 253)	Chronic, (*n* = 506)	*p*
Age (M ± SD), years	59 ± 14.8	58.9 ± 14.8	59 ± 14.8	0.931
Gender, men	288 (37.94%)	96 (37.94%)	192 (37.94%)	1
Rural	352 (46.4%)	113 (44.7%)	239 (47.2%)	0.503

M = Mean; SD = standard Deviation.

**Table 2:** Variation in Inflammation markers.

Marker	All (*n* = 759)	Acute (*n* = 253)	Chronic, (*n* = 506)	*p*
Lymphocytes (×10^9^/L)	1.96 ± 0.78	1.72 ± 0.81	2.08 ± 0.74	<0.001
Monocytes (×10^9^/L)	0.53 ± 0.36	0.6 ± 0.48	0.5 ± 0.28	0.001
Platelets (×10^9^/L)	268.40 ± 86.31	275.42 ± 100.32	264.89 ± 78.27	0.113
Neutrophils (×10^9^/L)	6.69 ± 4.05	9.28 ± 4.76	5.45 ± 2.96	<0.001
NLR	4.38 ± 3.36	7.02 ± 5.38	3.13 ± 3.09	<0.001
MLR	0.32 ± 0.34	0.44 ± 0.39	0.26 ± 0.21	<0.001
PLR	159.63 ± 93.49	194.06 ± 122.89	142.42 ± 68.5	<0.001

M = Mean; SD = Standard Deviation.

**Table 3:** Logistic regression model for investigated ratios.

Variables in the Equation									
		B	S.E.	Wald	df	Sig.	Exp (B)	95% CI for EXP (B)	
								Lower	Upper
Step 1 ^a^	NLR	0.257	0.037	48.621	1	0.000	1.293	1.203	1.390
	MLR	0.223	0.403	0.306	1	0.580	1.249	0.568	2.750
	PLR	0.001	0.001	1.066	1	0.302	1.001	0.999	1.004
	Constant	−2.163	0.210	106.156	1	0.000	0.115		

^a^. Variable(s) entered on step 1: NLR, MLR, PLR.

**Table 4:** AUC for investigated ratios.

Area Under the Curve	
Test Result Variable(s)	Area
NLR	0.806
MLR	0.609
PLR	0.666

**Table 5:** Cut off values for NLR.

Contingency Table—NLR				
		ACUTE vs. CHRONIC		
		1	0	Total
NLR	≥3.56	179	107	286
	<3.56	74	399	473
	Total	253	506	759

Note. Based on optimal cut-off (Youden’s Index).

**Table 6:** Diagnostic Accuracy -NLR.

		95% Confidence Interval	
	Result	Lower	Upper
Sensitivity	73.11%	66.61%	78.96%
Specificity	80.44%	76.47%	84.01%
Positive Likelihood Ratio	3.739	3.048	4.587
Negative Likelihood Ratio	0.334	0.266	0.419
Prevalence	32.02%	28.48%	35.73%
Positive Predictive Value	63.79%	58.94%	68.36%
Negative Predictive Value	86.40%	83.51%	88.85%
Accuracy	78.10%	74.75%	81.19%

**Table 7:** Variation in investigated markers between gangrenous and chronic cholecystitis.

Marker	Gangrenous (*n* = 145)	Chronic (*n* = 290)	*p*
Lymphocytes (×10^9^/L)	1.62 ± 0.75	2.14 ± 0.73	<0.001
Monocytes (×10^9^/L)	0.66 ± 0.53	0.57 ± 0.25	0.001
Platelets (×10^9^/L)	275.38 ± 107.76	255.83 ± 78.29	0.032
Neutrophils (×10^9^/L)	10.52 ± 4.99	4.77 ± 2.64	<0.001
NLR	8.23 ± 5.35	2.71 ± 1.77	<0.001
MLR	0.59 ± 0.4	0.35 ± 0.14	<0.001
PLR	209.33 ± 138.35	141.75 ± 72.57	<0.001

M = Mean; SD = Standard Deviation.

**Table 8:** Variation in investigated markers between gangrenous and phlegmonous cholecystitis.

Marker	Phlegmonous (*n* = 95)	Gangrenous (*n* = 145)	*p*
Lymphocytes (×10^9^/L)	1.8 ± 0.89	1.62 ± 0.75	0.014
Neutrophils (×10^9^/L)	7.94 ± 4.09	10.52 ± 4.99	<0.001
NLR	5.77 ± 4.33	8.23 ± 5.35	0.002
PLR	172.52 ± 97.14	209.33 ± 138.35	0.029

M = Mean; SD = Standard Deviation.

## Data Availability

The original contributions presented in the study are included in the article; further inquiries can be directed to the corresponding author.
